# A biogeographical appraisal of the threatened South East Africa Montane Archipelago ecoregion

**DOI:** 10.1038/s41598-024-54671-z

**Published:** 2024-03-12

**Authors:** Julian Bayliss, Gabriela B. Bittencourt-Silva, William R. Branch, Carl Bruessow, Steve Collins, T. Colin E. Congdon, Werner Conradie, Michael Curran, Savel R. Daniels, Iain Darbyshire, Harith Farooq, Lincoln Fishpool, Geoffrey Grantham, Zacharia Magombo, Hermenegildo Matimele, Ara Monadjem, Jose Monteiro, Jo Osborne, Justin Saunders, Paul Smith, Claire N. Spottiswoode, Peter J. Taylor, Jonathan Timberlake, Krystal A. Tolley, Érica Tovela, Philip J. Platts

**Affiliations:** 1 Department of Biological and Medical Sciences, Oxford Brookes University, Oxford, OX3 0BP UK; 2 African Butterfly Research Institute, P.O. Box 14308, Nairobi, 0800 Kenya; 3 Rede Para Gestão Comunitária de Recursos Naturais (ReGeCom), Maputo, Mozambique; 4 Natural History Museum, Cromwell Road, London, SW7 5BD UK; 5 Port Elizabeth Museum (Bayworld), P.O. Box 13147, Humewood, 6013 South Africa; 6 Mount Mulanje Conservation Trust, P.O. Box 139, Mulanje, Malawi; 7 Department of Nature Conservation Management, Faculty of Science, Nelson Mandela University, George, South Africa; 8 Department of Food System Science, Research Institute of Organic Agriculture (FiBL), Ackerstrasse 113, P.O. Box 219, 5070 Frick, Switzerland; 9 Department of Botany and Zoology, University of Stellenbosch, Private Bag X1, Matieland, 7602 South Africa; 10 Royal Botanic Gardens, Kew, Richmond, Surrey TW9 3AE UK; 11 Faculty of Natural Sciences, Lúrio University, Pemba, Mozambique; 12 Center for Macroecology, Evolution and Climate, Globe Institute, University of Copenhagen, Copenhagen, Denmark; 13 BirdLife International, The David Attenborough Building, Pembroke Street, Cambridge, CB2 3QZ UK; 14 Department of Geology, University of Johannesburg, Johannesburg, South Africa; 15 National Herbarium and Botanical Gardens of Malawi, Zomba, Malawi; 16 Herbarium, Instituto de Investigaçao Agraria de Moçambique, P.O.Box 3658, Maputo, Mozambique; 17 DICE, University of Kent, Canterbury, CT2 7NZ UK; 18 Wildlife Conservation Society, 163 Orlando Mendes Street, Maputo, Mozambique; 19 Department of Biological Sciences, University of Eswatini, Kwaluseni, Eswatini; 20 Mammal Research Institute, Department of Zoology & Entomology, University of Pretoria, Hatfield, South Africa; 21 Africa Bees Ltd, Belgrave House, 39–43 Monument Hill, Weybridge, Surrey KT13 8RN UK; 22 Botanic Gardens Conservation International (BGCI), 199 Kew Road, Richmond, Surrey TW9 3BW UK; 23 Department of Zoology, University of Cambridge, Cambridge, CB2 3EJ UK; 24 FitzPatrick Institute of African Ornithology, Department of Biological Sciences, University of Cape Town, Cape Town, South Africa; 25 Biological Sciences Department, University of Venda, Thohoyandou, South Africa; 26 Afromontane Research Unit and Department of Zoology & Entomology, University of the Free State, Bloemfontein, South Africa; 27 Biodiversity Foundation for Africa, East Dean, East Sussex UK; 28 South African National Biodiversity Institute, Kirstenbosch Research Centre, Claremont, Private Bag X7, Cape Town, 7735 South Africa; 29 Centre for Ecological Genomics and Wildlife Conservation, University of Johannesburg, Auckland Park, Johannesburg, 2006 South Africa; 30 Museu de História Natural, Praça Travessia do Zambeze, 104, Maputo, Mozambique; 31 Department of Environment and Geography, University of York, Wentworth Way, Heslington, York YO10 5NG UK; 32 BeZero Carbon Ltd, 25 Christopher Street, London, E2 UK

**Keywords:** Climate and Earth system modelling, Biodiversity, Biogeography, Conservation biology, Ecological modelling, Ecosystem ecology, Ecosystem services, Forest ecology, Palaeoecology, Tropical ecology, Ecology, Zoology, Ecology, Taxonomy, Evolution, Speciation, Adaptive radiation

## Abstract

Recent biological surveys of ancient inselbergs in southern Malawi and northern Mozambique have led to the discovery and description of many species new to science, and overlapping centres of endemism across multiple taxa. Combining these endemic taxa with data on geology and climate, we propose the ‘South East Africa Montane Archipelago’ (SEAMA) as a distinct ecoregion of global biological importance. The ecoregion encompasses 30 granitic inselbergs reaching > 1000 m above sea level, hosting the largest (Mt Mabu) and smallest (Mt Lico) mid-elevation rainforests in southern Africa, as well as biologically unique montane grasslands. Endemic taxa include 127 plants, 45 vertebrates (amphibians, reptiles, birds, mammals) and 45 invertebrate species (butterflies, freshwater crabs), and two endemic genera of plants and reptiles. Existing dated phylogenies of endemic animal lineages suggests this endemism arose from divergence events coinciding with repeated isolation of these mountains from the pan-African forests, together with the mountains’ great age and relative climatic stability. Since 2000, the SEAMA has lost 18% of its primary humid forest cover (up to 43% in some sites)—one of the highest deforestation rates in Africa. Urgently rectifying this situation, while addressing the resource needs of local communities, is a global priority for biodiversity conservation.

## Introduction

Tropical ecosystems of great antiquity are known to harbour exceptionally high levels of biodiversity and endemism^1^. In Africa, mountains typically host relict forests that are remnants of a widespread forest belt, which, prior to the uplift and long-term aridification of the East African plateaus, stretched across most of the continent^2–4^. As the global climate began to cool in the Early Oligocene, the pan-African rainforests began to fragment leading to significant forest reduction throughout the Miocene^4,5^. Much of the original forest in eastern Africa became confined to isolated montane patches that persisted due to orographic rainfall^4^. This caused forest-dependent species with low vagility to become trapped in upland refugia where moisture-laden trade winds maintained a relatively stable climate^6^. Subsequent climatic fluctuations through the Late Cenozoic disrupted gene flow between adjacent mountains^7^, driving allopatric speciation and establishing the unique biotic assemblages that now characterise African montane systems^4,8^.

To integrate conservation planning at relevant scales, it is helpful to recognise groups of mountains with shared geology, evolutionary history, and characteristic species assemblages as distinct ecoregions^9^. One of the best documented examples is the Eastern Arc ecoregion in Tanzania and Kenya^10^, long recognised as a global priority for conservation^11,12^. Much less known, however, are a series of granitic inselbergs (‘island mountains’) 600 km south of the Eastern Arc, stretching from southern Malawi across northern Mozambique. We propose that these inselbergs constitute an evolutionarily distinct montane ecoregion, characterised by high levels of endemism and ongoing threats from human activity.

Over the past century, numerous biological surveys have been undertaken on the mountains in southern Malawi. For example, Mt Mulanje, the second highest free-standing mountain in southern Africa (3002 m), is known to host a suite of endemic animal species^13,14^ and high levels of botanical endemism^15^, including the endemic Mulanje Cedar (*Widdringtonia whytei*)—Malawi's national tree^16^. In contrast, due to a protracted war for independence (1964–1974) followed by a civil war (1977–1992), the mountains in northern Mozambique remained largely unstudied by biologists until recently. Some mountains in northern Mozambique were surveyed in the late-19th through mid-twentieth centuries^17–20^, but only in the last 20 years have biological surveys begun to uncover the full extent of the region’s uniqueness. These more recent surveys began with ad hoc visits by ornithologists^21^ and herpetologists^22^. Then, following ecological surveys on Mt Mulanje, questions arose as to the degree of biological similarity, between Mt Mulanje and neighbouring inselbergs in Mozambique^23–25^. A series of scientific expeditions^26–32^ targeted sites in Mozambique above 1500 m, uncovering many species new to science and elucidating levels of shared endemism between these sites^33–53^. These findings prompted further surveys of other mountains in the region and resulted in new species descriptions, including many from Mt Mabu, which is now recognised to be the most extensive mid-elevation rainforest in southern Africa^54^.

Preliminary evidence for a new biogeographically distinct montane ecoregion was first proposed^54^ in 2014, and subsequently corroborated^55–58^. The name ‘South East Africa Montane Archipelago’ (SEAMA) was suggested in 2019, at the Annual General Meeting of the Transglobe Expedition Trust, Royal Geographical Society, London^59^, and formally proposed in 2022 at the 1st Southern African Mountain Conference in South Africa^60^. Here, we present a formal definition of the SEAMA ecoregion based on endemic species, geology, topography and climate, and place this in context through comparison with surrounding ecoregions. We synthesise and summarise all available records for taxa unique to the SEAMA, and thus report the degree of overlapping endemism across multiple taxa (plants, mammals, birds, reptiles, amphibians, crabs, and butterflies). Other taxonomic groups, such as most invertebrate groups, fungi and bryophytes await expert assessments and are therefore not included in our key estimates. Future assessments of these groups will invariably yield many more species new to science, thus further raising levels of endemism within the SEAMA. We assess potential drivers of speciation in different taxa, and suggest mechanisms for forest fragmentation that have produced the distinct ‘islands in the sky’ that harbour the unique biodiversity we see today^16,61^. Finally, we identify threats to the ecoregion and assess prospects for future conservation.

## Results

### Definition, extent and context

**Figure 1:**
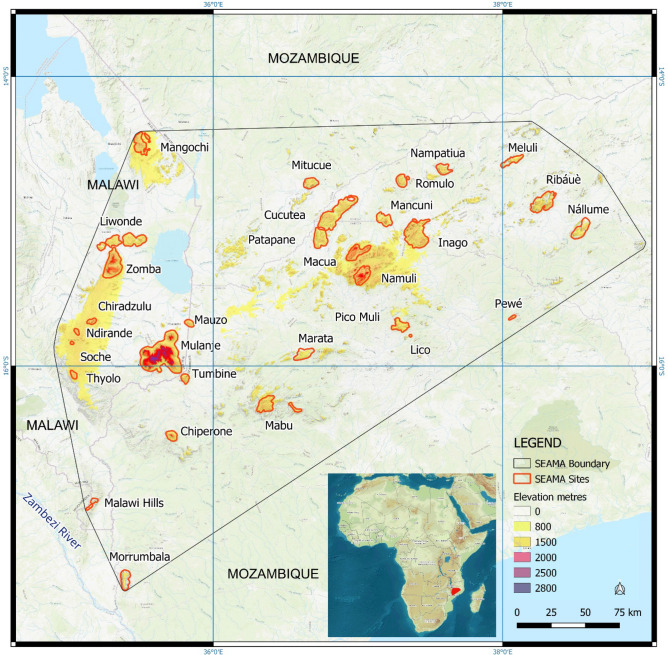
Location and extent of the South East Africa Montane Archipelago (SEAMA) showing core sites in red, and an outline boundary of the convex hull of the ecoregion (created using QGIS version 3.28.12 LTR https://qgis.org/en/site/).

We recognise at least 30 sites in the core of the SEAMA ecoregion (nine in Malawi, 21 in Mozambique; Fig. 1 and Table 1), each reaching an elevation of at least 800 m (elevation range is 500–3002 m) above sea level, and with high humidity (aridity index > 0.65). These sites host remnants of humid evergreen forest and upland grasslands, have ancient substrates, share similar climatic influences, and exhibit high levels of shared and single-site endemism compared with the ecosystems that directly surround them. More precisely, we define the SEAMA ecoregion as “a range of ancient granitic inselbergs in southern Malawi and northern Mozambique, climatically isolated by topography and trade winds, hosting humid evergreen forest, montane grassland and shrublands notable for their high levels of endemism across multiple taxa.”

**Table 1:** Details of the SEAMA core sites.

Site	Country	Lat DD Lon DD	Base contour (m)	Area (ha)	Summit (m)	Main habitats	% primary forest loss since 2000	# SEAMA endemics	Conservation status	Main threats
Mangochi FR	Malawi	− 14.465000 35.489722	1100	12,022	1733	Wet Forest Woodland Grassland	–	8	IBA KBA FR	Fire
Liwonde FR	Malawi	− 15.126944 35.497500	800	22,719	1613	Wet Forest Woodland	–	3	IBA KBA FR	Deforestation
Zomba / Malosa	Malawi	− 15.349444 35.285000	1100	20,712	2083	Wet Forest Woodland Grassland	–	34	IBA KBA FR	Deforestation Alien invasive plants
Chiradzulu	Malawi	− 15.69844 35.16554	1200	2320	1788	Wet forest Woodland Grassland	–	8	FR	Deforestation
Ndirande	Malawi	− 15.75524 35.05585	1200	1317	1599	Wet forest Woodland	–	8	FR	Totally deforested
Soche	Malawi	− 15.84162 35.02327	1300	522	1529	Wet Forest Woodland	–	8	IBA KBA FR	Totally deforested
Thyolo	Malawi	− 16.07378 35.04343	1200	2203	1464	Wet Forest Woodland	–	2	FR	Totally deforested
Malawi Hills	Malawi	− 16.92722 35.19028	700	2536	961	Wet Forest Woodland	–	1	IBA KBA FR	Almost totally deforested
Morrumbala	Mozambique	− 17.444167 35.395833	500	8368	1154	Wet Forest Woodland Grassland	8	3	KBA IPA	Deforestation Fire
Mulanje / Mchese	Malawi	− 15.949722 35.588056	900	62,995	3002	Wet Forest Woodland Grassland	7	115	IBA IPA KBA FR	Deforestation Fire Alien invasive plants
Tumbine	Mozambique	− 16.088257 35.801758	900	3319	1525	Wet Forest Woodland	14	4		Deforestation Fire
Mauzo	Malawi- Mozambique	− 15.70237 35.83582	800	2895	1486	Wet Forest Woodland	13	–		Deforestation
Chiperone	Mozambique	− 16.479167 35.712222	900	5005	2043	Wet Forest Woodland	14	11	IBA IPA KBA	Deforestation Fire
Mabu	Mozambique	− 16.298889 36.395556	1000	13,462	1699	Wet Forest Woodland Grassland	3	40	IBA IPA KBA	Fire Deforestation
Marata	Mozambique	− 15.92500 36.61513	700	8654	1276	Wet Forest Woodland Grassland	–	–		Deforestation
Patapane	Mozambique	− 15.135278 36.758333	800	12,353	1583	Wet Forest Woodland Grassland	21	–		Deforestation
Cucutea	Mozambique		800	29,729	1547	Wet Forest Woodland Grassland	–	–		Deforestation
Mitucue	Mozambique	− 14.73569 36.67015	700	6965	1460	Wet Forest Woodland Grassland	–	1		Deforestation
Macua	Mozambique	− 15.186389 36.988889	1400	11,359	2065	Wet Forest Woodland Grassland	19	–		Deforestation
Namuli	Mozambique	− 15.360278 37.058611	1400	12,012	2320	Wet Forest Woodland Grassland	30	82	IBA IPA KBA	Deforestation Fire
Pico Muli / Socone	Mozambique	− 15.732500 37.284722	900	7542	1556	Wet Forest Woodland Grassland	18	7		Deforestation Fire
Lico	Mozambique	− 15.791389 37.363333	800	308	1080	Wet Forest Woodland	2	8		Fire
Pewé	Mozambique	− 15.661111 38.073333	600	1036	1052	Wet Forest Woodland	–	1		Fire
Mancuni	Mozambique	− 15.008611 37.192778	800	8383	1755	Wet Forest Woodland Grassland	6	–		Deforestation
Inago	Mozambique	− 15.045000 37.396111	900	28,120	1769	Wet Forest Woodland Grassland	39	18	IPA KBA	Deforestation Fire
Romulo	Mozambique	− 14.715278 37.294167	700	6607	1537	Wet Forest Woodland Grassland	8	–		Deforestation
Nampatiua	Mozambique	− 14.652778 37.588611	700	6517	1571	Wet Forest Woodland Grassland	32	–		Deforestation
Meluli	Mozambique	− 14.568333 38.083889	800	6487	1466	Wet Forest Woodland Grassland	3	–		Deforestation
Ribáuè	Mozambique	− 14.874167 38.247500	700	18,168	1704	Wet Forest Woodland Grassland	35	27	IPA KBA FR	Deforestation Fire
Nállume	Mozambique	− 15.059167 38.546111	700	11,380	1440	Wet Forest Woodland	43	6	IPA	Deforestation
All CORE sites	–	–	–	336,015		–	18			

Base contours were used for site delineation, the results of which are shown in Fig. 1. Under recognition/reserve status: Important Bird Area (IBA); Key Biodiversity Area (KBA); Important Plant Area (IPA); Forest Reserve (FR). Dashes indicate no data for primary forest loss, and no data for SEAMA endemics.

Most mountains within the SEAMA were formed ca. 600–126 million years ago (Mya) as a result of igneous, rock-forming batholiths intruding into older, softer metamorphic rocks that subsequently weathered away^62,63^. The intrusions are mostly granitic and syenitic in composition^63,64^. Their mineralogy is characterised by low (typically < 10%) ferromagnesian mineral and high quartz and feldspar contents, rendering them relatively resistant to erosion. One aspect that contributes to this is the low degree of jointing in the rocks: SEAMA intrusions are characterised by homogeneous, randomly orientated crystals, which render them much less prone to jointing, water ingress and weathering, compared with, for example, some nearby gneisses (typically of planar fabric resulting from deformation and strain at an earlier time).

**Figure 2:**
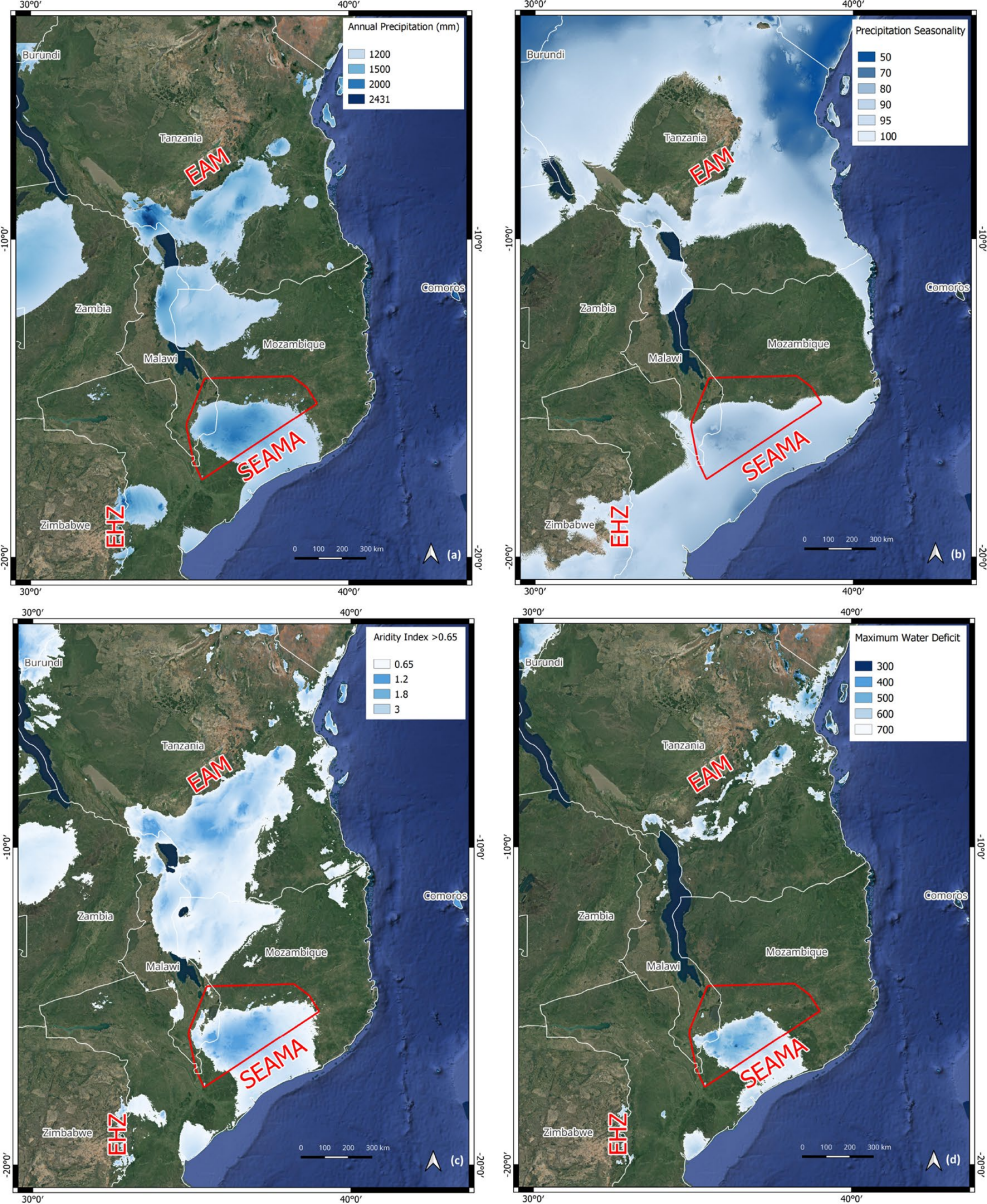
Contemporary macro-climatology of the South East Africa Montane Archipelago. Also labelled is the Eastern Arc Mountains (EAM) ecoregion to the north, and the Eastern Highlands of Zimbabwe to the southwest (EHZ). Annual rainfall (**a**) is measured in mm. Rainfall seasonality (**b**) is the coefficient of variation across months^65^. Aridity index (**c**) is the ratio of annual rainfall to potential evapotranspiration^66^ with values above 0.65 considered humid^67^. Maximum water deficit (**d**) is a measure of water stress defined across the most arid months of the year, with lower values conducive of potentially dense, evergreen canopy structure^68^. Bioclimatic layers were extracted from WorldClim version2 (https://www.worldclim.com/version2) and presented in QGIS version 3.28.12 LTR (https://qgis.org/en/site/).

The distinct nature of the SEAMA ecoregion is further clarified through contemporary patterns of rainfall and humidity, which clearly show the ecoregion falling under its own climatic envelope with a break to the north and connections to the coast (Fig. 2). This suggests that a defining factor is the south-east trade winds, funnelled up through the Mozambique Channel, carrying moisture to the mountains throughout the year. Mountains further north outside of the SEAMA (including Mts Njesi, Yao, and Mecula) are in the rain shadow of Madagascar, and historically have been more influenced by fluctuations in the water level of Lake Malawi. Further south (e.g., Mt Gorongosa), the air is cooler and so holds less moisture, especially in the dry season.

The SEAMA has distinctly higher annual rainfall and humidity, especially in the dry season, compared to surrounding regions (Fig. 2). This is similar to the Eastern Arc Mountains (EAM) and also the Eastern Highlands of Zimbabwe (EHZ). For plants, this equates to a shorter period of physiological stress, and hence a potentially longer growing season and the existence of mesic vegetation types such as humid evergreen forest. Moreover, since montane forests also extract moisture directly from the air via local orographic precipitation, the estimates of macroclimate presented here (Fig. 2) likely underestimate how much moisture the SEAMA forests receive relative to the lowlands^69^.

The convex hull of the ecoregion is defined by the farthest extents of the core sites identified through the selection criteria outlined by the methodology, plus other inselbergs at the periphery where elevations exceed 800 m and the aridity index exceeds 0.65 (humid climate). The interior of the convex hull also spans arid lowland habitats which, during cooler climes, would have periodically connected the now isolated inselberg habitats, as well as smaller inselbergs that no longer support humid evergreen forest, either due to lower elevation (< 800 m) or because the forests on these sites have already been cleared by humans (some of the core sites have also been largely deforested, e.g., Chiradzulu, Thyolo).

Accordingly, the SEAMA ecoregion has a core area (sum of the areas of the 30 individual sites) of 336,200 ha with a total extent of occurrence (convex hull) close to 10 million ha (Table 1). In relation to the WWF Global 200 ecoregions^9,70,71^, the SEAMA has a smaller core area than some of its neighbours, such as the Eastern Arc Montane Forests EAM (2.4 million ha), the Southern Rift Montane Forest-grassland Mosaic SRMFM (3.3 million ha), the East African Montane Forest ecoregion EAMF (6.5 million ha), and the Ethiopian Montane Forests EMF (24.9 million ha). However, it is a similar size to other mountain ecoregions such as the East African Montane Moorlands EAMM (330,000 ha) and the Knysna-Amatole Montane Forests KAMF (310,000 ha). The SEAMA incorporates the South Malawi Montane Forest-grassland Mosaic, which represents the Malawi component on this larger SEAMA ecoregion, and the Mulanje-Namuli-Ribáuè sub-Centre of Plant Endemism^55^. It also forms part of the Africa-wide Afromontane Archipelago botanical Centre of Endemism^2^. In relation to Key Biodiversity Areas (KBAs) in the Eastern Afromontane Biodiversity Hotspot^72^, the SEAMA is located between the Northern Lake Nyassa Catchments (Southern Rift montane forest-grassland mosaic) and the Chimanimani-Nyanga Catchments (Eastern Zimbabwe montane forest-grassland mosaic). The SEAMA ecoregion encompasses nine Important Bird Areas (IBAs), eight Important Plant Areas (IPAs) from Mozambique^73^ (yet to be determined for Malawi), and 12 Key Biodiversity Areas (KBAs) in Malawi and Mozambique^74^ (Table 1).

### Comparative ecoregion endemism

**Figure 3:**
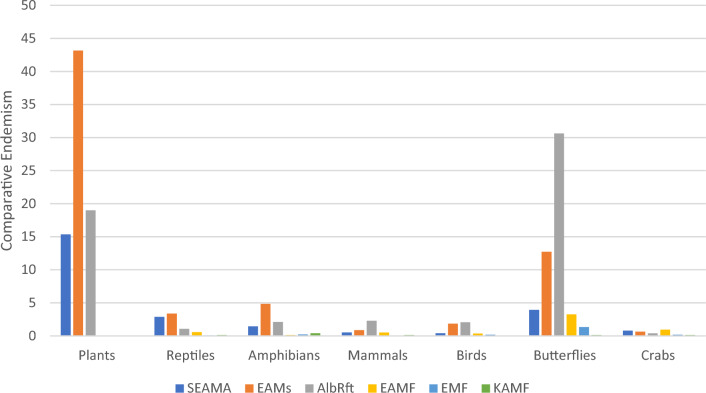
Comparative ecoregion endemism according to the species-area function. Within each taxonomic group and ecoregion, we plot the number of endemics divided by A^z^, where A is the core area (ha) of the ecoregion and z = 0.25 is taken as a representative value of the slope of the species-area curve. This yields a metric of endemism to estimate the number of endemic species per hectare, which diminishes the impact of larger areas.

Compared to five neighbouring mountain ecoregions, levels of endemism in the SEAMA are higher than most in the taxonomic groups surveyed. Notably the number of strictly endemic reptile species is higher than the much larger Albertine Rift (AlbRft) ecoregion (Table 2). When area is taken into consideration using a species-area function (see “Methods”), the SEAMA is a close third in ecoregion endemism to the EAMs and the AlbRft across most taxa, and second in comparative endemism for reptiles and crabs (Fig. 3).

**Table 2:** Number of strictly endemic species by taxonomic group in mountain ecoregions in east and southern Africa.

Ecoregion	Plants	Reptiles	Amphibians	Mammals	Birds	Butterflies	Crabs
SEAMA	117	22	11	4	3	30	6
EAM	536	42	60	11	23	158	8
AlbRft	341	19	38	41	37	550*	7
EAMF	DD	9	2	8	6	52*	15
EMF	DD	2	5	1	4	30*	4
KAMF	0	1	3	1	0	0	1

*DD* Data Deficient, *Expert judgement (ABRI).

*Expert judgment was engaged to assess the endemic butterflies for three of the neighbouring ecoregions based on published and unpublished data by the Executive Director of the African Butterfly Research Institute (ABRI). Due to the very comprehensive butterfly database at ABRI, this was considered acceptable and reasonably accurate in this instance. ABRI houses the largest global collection of African butterflies.

### Relative survey effort per site

**Figure 4:**
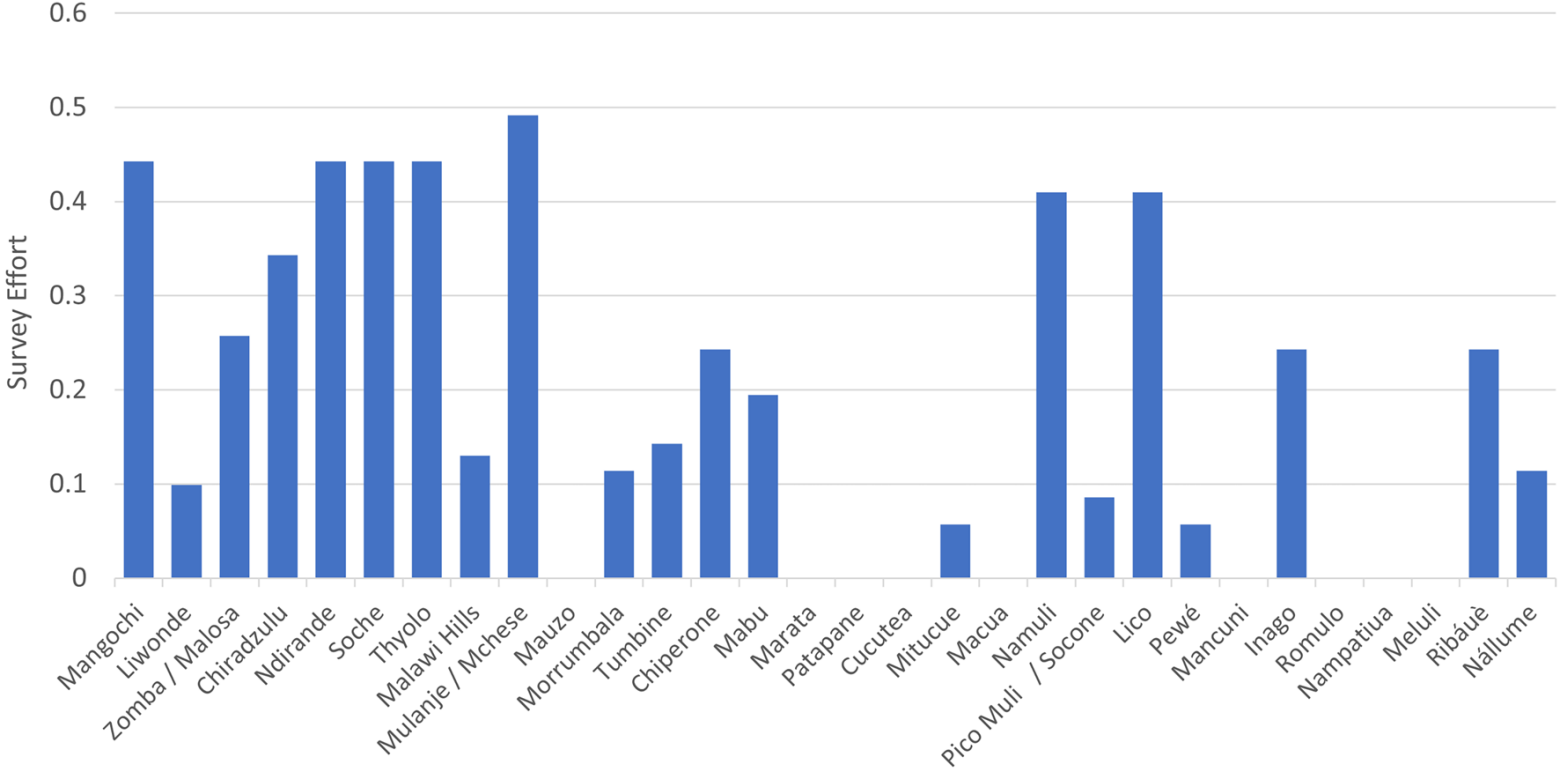
Relative survey effort across core sites in the SEAMA. Bars plot a comparative measure of sampling intensity, relative to the area of each site^75^. Score varies between 0 and 1, where 0 is the minimum (no sampling at all) while 1 is the maximum effort possible to obtain.

Historically, relative survey effort (defined as sampling intensity relative to mountain blocks and site area^75^, see “Methods”) has been much greater in Malawi than in Mozambique (Fig. 4). Overall, the mean relative survey effort for the whole of the SEAMA is 0.22, while the Malawi sites have a mean relative survey effort of 0.41, compared to 0.12 for Mozambique. The most comprehensively surveyed site in Malawi is Mount Mulanje, followed by Mangochi, Ndirande, Soche, and Thyolo. In Mozambique, the most comprehensively surveyed sites are Mabu, Namuli, and Lico, and nine Mozambique sites have never been scientifically surveyed.

### Biological uniqueness

**Figure 5:**
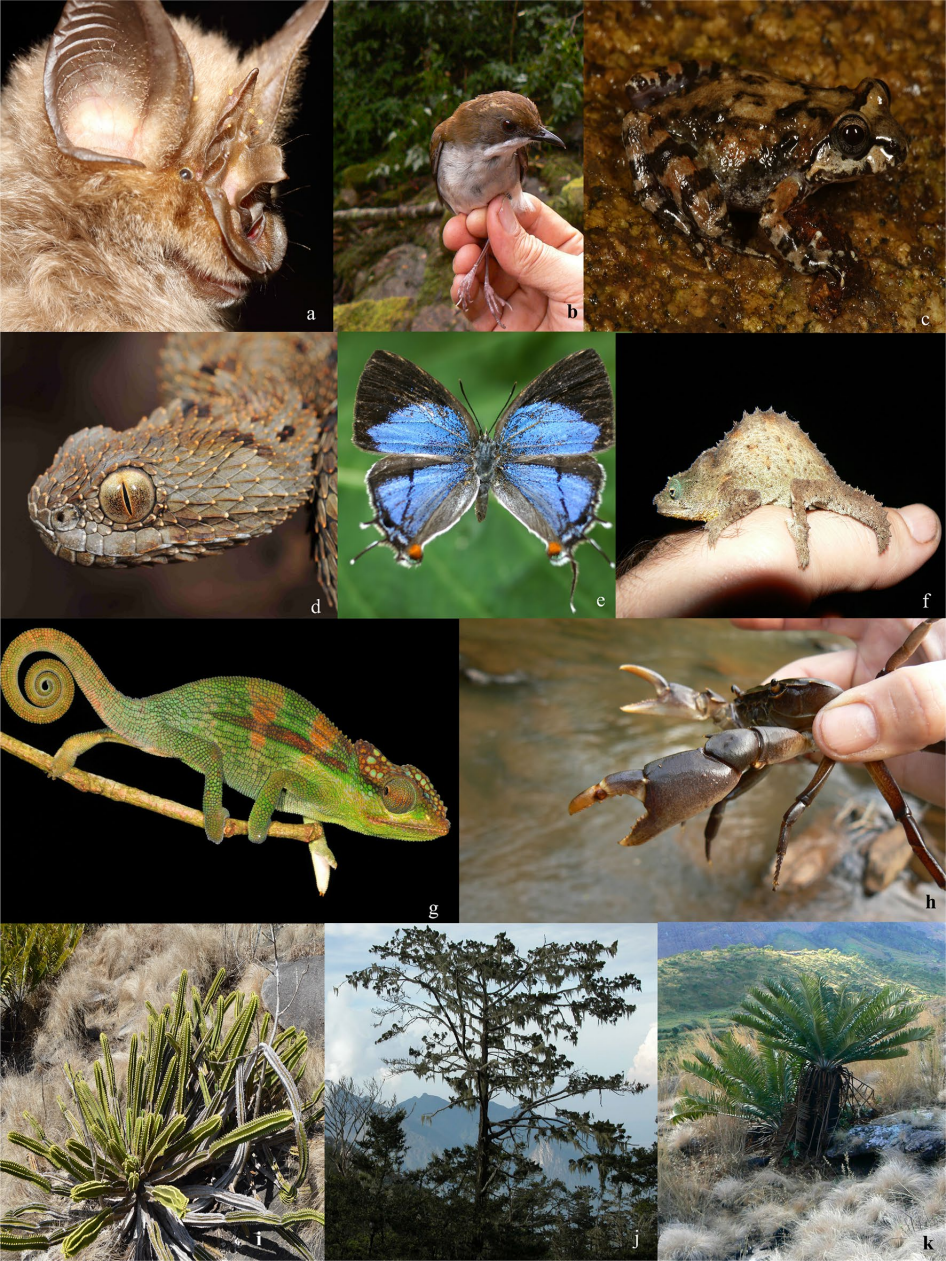
Examples of SEAMA endemics. (**a**) *Rhinolophus mabuensis* (AM), (**b**) *Chamaetylas choloensis* (JB), (**c**) *Nothophryne inagoensis* (WC), (**d**) *Atheris mabuensis* (WRB), (**e**) *Epamera malaikae* (TCEC), f) *Rhampholeon maspictus* (JB), (**g**) *Nadzikambia baylissi* (WRB), (**h**) *Maritonautes namuliensis* (JB), (**i**) *Euphorbia mlanjeana* (ID), (**j**) *Widdringtonia whytei* (JB), (**k**) *Encephalartos gratus* (JB).

The SEAMA hosts endemic genera and species assemblages (Fig. 5a−k), which characterise the ecoregion (Table SI1). Currently, 217 endemic taxa (192 strictly endemic species, plus 25 subspecies and races) are recognised, with a greater number of endemic plants than animals (127 *vs* 90 taxa, Table 3). The number of endemic taxa is notably high for such a limited geographic area. We expect the number of known endemics, especially amongst the fauna, to grow substantially with additional research, given the low sampling effort for some groups (e.g., although reptiles have been relatively well surveyed, small mammals have not)^76^. Overall, of the groups surveyed, levels of endemism are proportionally highest among reptiles, amphibians, mammals, crabs, and butterflies. Most endemic animals are forest specialists, as opposed to endemic plants which are typically restricted to high-elevation grasslands and lithophyte communities. Taxonomic influences are evident from all surrounding ecoregions, however there is greater influence from the north (Tanzania) amongst the faunal groups, which suggests a historical continuous humid forest belt stretching from eastern to southern Africa.

**Table 3:** Number of endemic taxa known from the South East Africa Montane Archipelago and their conservation status.

Taxon	Endemic species	Endemic subspecies	Endemic varieties/races	Total endemic taxa	IUCN, VU, EN, CR (%)
Plants	117	7	3	127	38 (30%)
Mammals	4			4	2 (50%)
Birds	3	5		8	4 (50%)
Reptiles	22			22	12 (55%)
Amphibians	11			11	7 (64%)
Crabs	6			6	0
Butterflies	30	9		39	7 (18%)
TOTAL	193	21	3	217	

The last column also indicates the percentage of species considered Threatened (VU, EN, CR) on the IUCN Red List (accessed September 2022).

### Plants

A subset of the SEAMA sites has recently been recognised as forming a distinct botanical sub-centre of endemism^55^, part of the Africa-wide Afromontane archipelago-like Centre of Endemism, as defined by White^2^. Among the best studied sites in the SEAMA are Mts Mulanje^16,77,78^, Zomba^79,80^, Namuli^30,34,81^, Mabu^29,54,82^, Chiperone^31,34^, and Ribáuè^55,83^. Plants account for 117 strictly endemic species and a total of 127 endemic taxa (Table 3, Fig. 5i−k, and Table S1), with the main single areas being Mts Mulanje (48 endemic taxa) and Namuli (19 endemic taxa) (Table S1). Of those assessed for the IUCN Red List, 37 (29%) are listed as threatened (VU, EN, CR). Approximately 69% of endemic plant taxa occur on rocky crags and/or grassland, and only 28% are limited to forest or forest margins. Only three endemic plant taxa show any link to woodland. It is likely that endemic plant taxa remain to be scientifically discovered in the SEAMA from sites currently not surveyed, and from the forests and forest margins of Mt Mabu.

### Mammals

Mammals account for four strictly endemic species (Table 3 and Table S1). All are small (< 250 g in mass), and all are forest-dependent. Of those assessed, two (50%) are listed as threatened (VU, EN, CR) (IUCN 2022), while the remainder await formal description. Given narrow ranges and high rates of forest loss, the remaining two mammals will likely be considered Threatened by the IUCN once they are formally described. Because of the relatively low sampling effort for small mammals, the expectation is that many species remain scientifically undiscovered, and so diversity and richness in this group is likely to be significantly higher than is currently known^84^.

### Birds

Birds account for eight endemic taxa, three strictly endemic species and five endemic subspecies (Table 3 and Table S1). All endemic taxa are forest-dependent, and most are considered highly threatened. Four (50%) are listed as Near Threatened (NT) or threatened (VU, EN, CR) on the IUCN Red List. Sampling effort has been good on some mountains (Mts Mulanje, Zomba, Mangochi, Liwonde, Namuli, Mabu, and Chiperone) but most of the other mountains are yet to be surveyed by ornithologists. However, the chance of finding new endemic bird taxa is low, since the remaining forest fragments across unsurveyed sites are generally too small to expect new ornithological scientific discoveries.

### Reptiles

There are 22 strictly endemic reptile species (Table S1). Of these, 19 (86%) are forest-dependent species, and the others occur mainly in upland grasslands and on rock faces. As they predominantly occur in forest, SEAMA’s endemic reptiles are highly threatened by habitat loss. Of those assessed for the IUCN Red List, 12 (55%) are listed as Near Threatened (NT) or threatened (VU, EN, CR), while at least six species await formal description, after which they will almost certainly qualify for an IUCN Threat category. Sampling effort has been adequate for certain sites (Mts Mulanje, Mabu, Namuli, Chiperone, Ribáuè), but many others remain unsampled. Given that new species have been scientifically discovered on each of the surveyed mountains, it is likely that other mountains harbour undescribed endemics, many of which will probably be considered threatened by ongoing forest loss.

### Amphibians

There are 11 strictly endemic amphibian species (Table 3 and Table S1). Approximately half of these occur in forest and of those assessed for the IUCN Red List, seven (64%) are listed as Near Threatened (NT) or threatened (VU, EN, CR). Sampling effort has been adequate for some mountains (Mts Mulanje, Mabu, Namuli, Ribáuè) although some fossorial species are probably under-sampled. There are many other mountains that remain poorly sampled or unsampled. New species have been scientifically discovered on some of the recently surveyed mountains^32,46,85^, suggesting that further efforts on other mountains will likely reveal new species or new populations of endemic amphibians, and increased representation on the IUCN Red List.

### Freshwater crabs

There are six strictly endemic freshwater crab species to the SEAMA ecoregion, two of which are undescribed species (Table 3 and Table S1). Currently, most of the recognised endemic diversity occurs in the newly established *Maritonautes* group (Fig. 5h). Four of the endemic species are found inside forest (67%), although none have yet been assessed for their IUCN threat status. Given that new species have been scientifically discovered on several mountains, there is a high likelihood of finding new species of freshwater crab on other mountains.

### Butterflies

There are 39 endemic butterfly taxa in the SEAMA ecoregion including 30 strictly endemic species (Table 3, Fig. 5e, and Table S1). The majority (75%) occur in forest and given the degree of forest loss due to slash and burn agricultural expansion^28–31^; these species are under threat. Of those assessed for the IUCN Red List, seven (18%) are listed as threatened (VU, EN, CR). Sampling effort has been adequate in certain sites (Mts Mulanje, Mangochi, Zomba, Mabu, Namuli, and Lico), with single collecting visits made to some other sites (Mts Chiperone, Socone, Pewé, Nállume), leaving many others that remain scientifically unexplored. Given that new species have been scientifically discovered on most mountains, there is a high likelihood of finding more species of butterflies new to science.

## Discussion

### Origins, connections, and divergence

Many of the plant endemics, including several genera, have their closest relatives in the Southern Afromontane region (stretching from southern Tanzania to the Western Cape province of South Africa), as opposed to elsewhere in East Africa. Although there are some links between the endemic plants of Mt Mabu and the Southern Highlands of Tanzania (e.g., *Helixanthera schizocalyx*), there appear to be more links with areas to the south, such as the Nyanga-Chimanimani area, while a few appear to be more strongly linked to the East African coastal belt. Others have more general affinities with southern African forms (especially those of drier formations) or even the wider Afrotropics.

Within vertebrates, the closest relatives of the endemic mammals are mainly East African (e.g., with *Rhinolophus mabuensis* (Fig. 5a) close to *R. hildebrandtii*). Amongst birds, the affinities are mainly with Tanzania to the north and Zimbabwe to the west. For reptiles, the closest relatives are to the north or west depending on the genus, and so taxonomic influences are from all surrounding regions. The closest relatives of endemic amphibians are mainly from east Africa, with just two species associated with southern Africa.

Amongst invertebrates, the closest relatives of the endemic butterfly fauna are East African (88%), with just four species with affinities to central and southern Africa. Therefore, the influences are predominantly northern. The closest relatives of endemic crabs are mainly East African, with 40% connected to Zimbabwe. Therefore, the influences are from the north and the west. Divergence time estimation for endemic freshwater crab species suggest cladogenic activity was initiated during the late Miocene and continued to the Pliocene^50,86^.

Species-level divergences for some of the genera in the SEAMA have been estimated, with divergence estimates ranging from relatively recently (e.g., Plio-Peistocene) to fairly ancient (e.g., Mid-Miocene), Table SI2. As of yet, no common patterns have emerged, in part due to information on the timing of divergence being lacking for most genera. Nevertheless, for some of the small mammal species, especially the bats (*Rhinolophous mabuensis*, Fig. 5a), species-level divergences are relatively recent, e.g., 1–2 Mya. The divergence dates between butterfly species have yet to be examined, although a new species of *Cymothoe* diverged from sister taxa approximately 4 Mya^49^. Within the freshwater crabs (*Potamonautes* and *Maritonautes*) species-level divergence dates are estimated at 2.5–8 Mya^50^. In contrast, estimated divergence dates for reptiles and amphibians are more ancient, with the earliest diverging lineages dating to the Mid-Miocene. For example the bush viper *Atheris mabuensis* (Fig. 5d), the sole representative of the genus from the SEAMA, diverged from its East Africa sister taxa around 15 Mya^33^. The pygmy chameleons (*Rhampholeon* spp.) are better represented in the SEAMA, with eight of the 25 described species (plus probably several undescribed) occurring on isolated mountains. Six of these form a monophyletic clade, having diverged from the East African species more than 10 Mya^37,87^. The two remaining *Rhampholeon* species are sister to East/Central African species and likely diverged even earlier. Similarly, species-level diversification within the SEAMA endemic amphibian genus *Nothophryne* (Fig. 5g) are ancient, between 7.5 and 18 Mya^85^. Unlike the chameleons, *Nothophryne* shares a common ancestor with southern African taxa, not East African^86^ suggesting that the SEAMA has linkages to both East and southern Africa. Although additional studies are needed to assess common patterns, the reptile and amphibians essentially exhibit some of the oldest species known from the SEAMA (e.g., *Nothophryne broadleyi*, 18.65 Mya; *Atheris mabuensis*, 15 Mya).

Amongst the chameleons, *Rhampholeon* and *Nadzikambia* (Fig. 5f,g), most species are endemic to a single mountain, apart from *R. tilburyi*, which has been recorded from at least five mountains (and is therefore a SEAMA regional endemic). Mts Namuli, Inago, Mabu, Chiperone, Mulanje and the Malawi Hills all have an endemic *Rhampholeon*, with an endemic *Nadzikambia* recorded for each of Mabu (Fig. 5g), Chiperone, and Mulanje. Chameleons on these inselbergs probably shared a common ancestor in the Mid-Miocene^37^. Phylogenetic analysis has shown that *R. bruessoworum* is the oldest diverging lineage, dated to the Mid or Early-Miocene (ca. 11–30 Mya). This may have been followed by the isolation of *R. platyceps* on Mt Mulanje and *R. tilburyi* on Mts Namuli, Pico Mulli, Nallume, Ribáuè, and Pewe in the Mid to Late Miocene. Divergence times for *Rhampholeon* from Mts Mabu (*R. maspictus*, Fig. 5f), Chiperone (*R. nebulauctor*), and the Malawi Hills (*R. chapmanorum*) are more recent (4–9 Mya) within the Late Miocene or Early Pliocene, suggesting these forests remained connected until most recently^37^. The two most closely related species are *R. nebulauctor* from Mt Chiperone and *R. chapmanorum* from Malawi Hills, which are just 75 km apart and may have been connected through the Late Pliocene^37^. A recent phylogenetic study^50^ showed that initial diversification with the SEAMA endemic crabs (*Potamonautes* and *Maritonautes*) dates to just over 7 Mya, with species-level diversification mainly within the Pliocene, e.g., *Maritonautes namuliensis*, 5.4 Mya (Fig. 5h); *Potamonautes mulanjeensis*, 2.84 Mya; *Maritonautes licoensis*/*Maritonautes choloensis*, 2.5 Mya.

Overall across taxa, dating estimates suggest that the initiation of allopatric speciation through vicariance of forest patches began in the mid-Miocene for some groups but with other groups diversifying more recently. Thus, vicariance events do not appear to be coeval suggesting the SEAMA taxa have a complex evolutionary history that has melded to form the rich diversity of the region. For example, species-level divergences within the amphibian genus *Nothophryne* from Mts Mulanje, Namuli and Ribáuè can be dated to approximately 18 Myr, 13 Myr and 7 Myr, respectively^85^ and the one SEAMA endemic clade of *Rhampholeon* shows a similar pattern. Thus, vicariance of the forests on these mountains may have been sequential, resulting in isolated populations that diverged in allopatry. Forest specialists or non-vagile species may have been prone to early vicariance events, becoming isolated in forest patches. The overall patterns to date suggest that Mt Mulanje became isolated first, followed by the eastern SEAMA mountains such as Mts Namuli, Mabu, Chiperone and Mt Ribáuè. Clearly, the high species richness that defines the SEAMA was formed as an amalgamation of both ancient and also recently diverged lineages (crabs, butterflies, and bats), suggesting the processes that contribute to speciation and diversification have been ongoing over tens of millions of years.

Dated phylogenetic analyses show that the earliest SEAMA lineages date back to the Mid- or Early-Miocene. Therefore, we can assume that this ecoregion’s forest, started to become isolated from at least the Mid-Miocene. Given that species-level diversification is evident throughout the Mid-to-Late Miocene and into the Plio/Pleistocene, vicariance of these forests would have continued with subsequent isolation of species. These dates are similar to published data on the breakup of the continuous forest belt that covered this part of Africa at this time^2^. Throughout the Miocene, despite periods of wet and dry cycles, the climate became drier overall, resulting in a reduction of forest and an increase in woodland and open grassland. Forests in eastern and southern Africa were consequently confined to areas with higher precipitation, and especially to mountains^1,11,78,88^, resulting in a fragmented forest landscape with upland grasslands in which endemic species emerged as a result of the repeated isolation.

### A threatened ecoregion

The major cause of montane forest loss in the SEAMA is slash and burn shifting agricultural practices, typically used for subsistence food production by local communities, along with charcoal production, for household cooking and as a source of revenue (sold on for use in urban areas)^16^. The fertility of the forest soil is valued, which results in smallholder plots inside or on the margins of forest being cleared for crops (especially maize, cassava, and Irish potatoes). Fire from such agriculture affects the forest edges around these cleared plots. Most of these forest patches are naturally small (< 1000 hectares) and edge effects therefore have a disproportionate impact on their ecological integrity. Where forest edges are proportionally high and degraded, the intact forest interior can be compromised due to drying effects. The upland grasslands are also threatened by increases in fire frequency, sometimes associated with flushing animals into traps. Conservation agriculture practices and sustainable alternative livelihoods are required to address these threats throughout the SEAMA ecoregion.

Official protection mechanisms vary between Malawi and Mozambique, as well as among the individual mountains. In Malawi, all the SEAMA sites are gazetted as national Forest Reserves under the management of the Department of Forestry, although this does not seem to afford adequate protection for either the forests or their natural resources, as regulated and unregulated deforestation for timber extraction and charcoal production is rife^16^. Thus, the wet forest on Thyolo Mountain (the type locality for several endemic taxa, including birds such as *Chamaetylas choloensis* (Fig. 5b) and the endemic race *belcheri* of *Cryptolybia olivacea*) was eliminated approximately 20 years ago, with the exception of a small (0.27 ha) forest patch on private land. The same fate affected Ndirande Mountain (in the 1990s) and Soche Mountain (2010s), as also most of Chiradzulu Mountain and the Malawi Hills^89^. In Mozambique, only one of the SEAMA sites, Mt Ribáuè (which includes Serra Mpàluwé) is a gazetted Forest Reserve. However, most forest reserves were established to regulate the harvesting of timber, not for establishing protected areas in terms of conservation^90^, and therefore Mt Ribáuè lacks any tangible, formal protection. Although all other sites lack any formal protection through national legislation, a project is currently underway to declare Mt Mabu as a ‘community-conservation’ protected area. Some SEAMA sites in Mozambique, such as Mt Lico, are protected due to their natural inaccessibility, while some others, such as Mt Mabu and Mt Pewé have remained in relatively good condition, possibly because these forests have spiritual value to the local communities. Overall, however, the majority of SEAMA sites lack meaningful national protection in contrast to conservation initiatives implemented in neighbouring ecoregions, such as the Eastern Arc Mountains in Tanzania.

At the level of taxonomic groups, extinction risks vary according to their reliance on the forest or upland grassland habitats (Table 2). Most of the small mammals, birds, reptiles, and amphibians, are forest-dependent and therefore the proportion of threatened species is generally high due to the acute forest destruction on most mountains. Therefore, many of these forest-dependant species are listed as Near Threatened (NT) or threatened (VU, EN, CR) on the IUCN Red List, for example the *Atheris mabuensis* (Fig. 5d), *Rhinolophus mabuensis* (Fig. 5a), *Apalis lynesi*, *Rhampholeon chapmanorum*, and *Paraxerus vincenti*—see Supplementary Material for a complete list. For the endemic plants (e.g., Fig. 5i−k) the majority (69%) are found in open habitat which are threatened to varying degrees by increased fire frequency^30,91^. However, forest plant species such as *Helixanthera schizocalyx*, the two new *Polysphaeria* species from Mts Ribáuè and Mabu^42^, and the forest tree *Faurea racemosa*, are all listed as Endangered on the IUCN Red List.

The greatest threat for the majority of sites is deforestation and increased fire frequency to the upland grasslands. Within the area defined (convex hull) for the SEAMA ecoregion, approximately 18% of primary humid forest above 800 m in elevation was lost between the years 2000 and 2022 (Table 1). This, for the same period, is far greater than in other African mountain ecoregions that contain primary humid montane forest^91^, including the Eastern Arc, Cameroonian Highlands, and Mt Cameroon and Bioko montane forests (< 5%), and the Albertine Rift and Guinean montane forests (ca. 10%). Therefore, the SEAMA is considerably more threatened by the rate of primary humid forest loss. Within the SEAMA, the extent and timing of forest loss varies between sites, with some such as Chiradzulu, Ndirande, and Thyolo Mountains in Malawi having lost all their forest cover prior to the year 2000, while the Malawi Hills are estimated^89^ to have lost nearly 80% of forest cover since the 1980s. Several other sites have suffered severe declines in the period 2000 to 2022 (Table 1), including Mts Nállume 43%, Inago 39%, Ribáuè 35%, Namuli 30%, Socone 18% and Chiperone 14%. The sites with the least loss of primary humid forest over the last 22 years are Mts Meluli and Mabu (both 3%), and Mt Lico (< 2%), this latter figure is because the Mt Lico forest is essentially inaccessible.. Note that these estimates only account for loss of primary humid forest, not secondary forest, or woodland, such that the actual habitat loss including the lower mountain slopes is likely to be much higher^91–93^. More generally, rates of montane forest loss in Mozambique, at close to 30% since 2000, are among the highest in tropical Africa^91^.

## Conclusions

An ecoregion has shared biotic and abiotic characteristics that distinguish it from surrounding areas^9,70^. In the case of the SEAMA, a distinct climatic envelope clearly characterises a specific range of mountains, and defines its boundaries. Within these boundaries, we find unique species assemblages, characterised by an abundance of endemic lineages. Although survey effort has improved in recent decades for the SEAMA, compared to other neighbouring regions (e.g., Eastern Arc Mountains) its Relative Sampling Effort is still comparatively low (Table 1). Despite this, the SEAMA has more strictly endemic reptile species than the much larger and better-known Albertine Rift ecoregion. Where biological sampling has been relatively high within the SEAMA, e.g., Mt Mulanje, significant levels of endemism have been found, with a total of 48 endemic plant species (modified from Strugnell^15^) and a range of endemic fauna^13,14,16^.

The South East Africa Montane Archipelago (SEAMA)^11,21,78,88^ might be Africa’s newest and most threatened ecoregion with one of the highest deforestation rates (18%); however, the evidence base now exists for its global recognition as a priority site for conservation.

## Methods

### Definition, extent and context

For the purposes of data collation and mapping, we bounded the ecoregion in two ways. First as a collection of core sites, each individually named and spatially delimited. These are the sites of greatest known biological interest. Second, we defined a convex hull around the core sites, plus other inselbergs with the requisite elevation (> 800 m) and humidity (aridity index > 0.65). Thus, the convex hull encompasses the core sites, other inselbergs (with or without forest remaining), as well as lowland connections in between. We defined ‘endemic taxa’ as those found only within the convex hull of the ecoregion and associated with one or more of the 30 core sites (as per Fig. 1 and Table 1). Our definition of endemic taxa includes strictly endemic species, subspecies, and races. Our analysis is based on the taxonomic groups we have listed. It is recognised that levels of endemism will further increase when additional taxonomic groups are sampled.

We bounded the core sites as close to the base as possible, selecting the lowest 100-m elevational contour that distinguishes only the target features. Due to variation in base elevations across the SEAMA extent, different contours were appropriate for different sites (Table 1). The chosen contours were converted to polygons, buffered by 500 m and then smoothed to aggregate features and simplify the topology. We used digital elevation data from the Shuttle Radar Topography Mission (SRTM) v3 at 1 arc-second (~ 30 m) spatial resolution, gap-filled using a bilinear resampling of the 3 arc-second version of the same product (which has no gaps).

For the aridity index, we used the 30 arc-second (~ 1 km) surface provided by CGIAR-CSI^66^. The aridity index is defined as the ratio of mean annual precipitation to potential evapotranspiration, where values < 0.2 are indicative of an arid or hyper-arid environment, 0.2–0.5 semi-arid, 0.5–0.65 dry sub-humid, and > 0.65 humid^67^. Evapotranspiration was based on the FA0-56 Penman–Monteith Reference Evapotranspiration equation, using rainfall and temperature estimates from WorldClim version 2^65^. In Fig. 2, we mapped this aridity index, and three other climatic variables that we expect to correlate with ecoclimatic stability^94^: annual rainfall (BIO12 in WorldClim2), rainfall seasonality (BIO15) defined as the coefficient of variation in rainfall across months, and maximum water deficit, a measure of dry season water stress^67^. We computed the maximum water deficit across consecutive months that experience rainfall < monthly ET0, over which the shortfall in rain was accumulated^68^.

### Comparative ecoregion endemism

To place SEAMA (336,200 ha) in context, we compared the total number of strictly endemic species from various taxonomic groups with five other mountain ecoregions in east and southern Africa that are found within the Tropical & Subtropical Moist Broadleaf Forests category of the Afrotropical ecoregions according to the WWF Terrestrial Ecoregions Global 200 assessment. These are the Eastern Arc Mountains forests (EAM) at 2,380,000 ha; the Albertine Rift (AlbRft) at 10,390,000 ha; the East African Montane Forests (EAMF) at 6,563,700 ha; the Ethiopian Montane Forests (EMF) at 24,930,200 ha; and the Knysna-Amatole Montane Forests (KAMF) at 310,800 ha. In this context, in all taxonomic groups surveyed, ‘endemic species’ refer to strict endemic species only, while ‘endemic taxa’ refer to strict endemic species, endemic subspecies and endemic races.

Although direct comparisons between the absolute number of strictly endemic species can be made (Table 2), there are orders of magnitude differences in area between these ecoregions. To account for these differences in area, we applied a species-area function to estimate the number of endemic species per hectare^95^ by dividing richness (strict endemic species) by A^z^, where A is the core area (ha) of the ecoregion and z is the slope of the species-area curve (= 0.25), which diminishes the impact of larger areas.

### Relative survey effort per site

We estimated relative survey effort per site using a composite index that takes into account the area of each SEAMA site (A), and sampling intensity per site (SI)^75^. Survey effort reflects the amount of time and resources allocated to assess the biophysical aspects of each site within SEAMA. As such it allows comparison of sampling effort between ecoregions which adds context to the known levels of endemism and the likelihood of increasing these levels. We scored sampling intensity using a 0–1 scale as follows: 0 = no known studies in that site; 0.2 = limited and non-systematic survey over few localities in the site; 0.5 = systematic survey over many localities in the site; and 1 = extremely thorough and systematic survey covering all habitats in the site.

$$\sum\limits_{0}^{i} {(A_{i} \cdot SI_{i} )} /\sum\limits_{0}^{i} {Ai}$$

We adapted the original formulae and calculated the mean sampling intensity (SI) for all the taxonomic groups studied at each site. In our case *I* indicates the number of mountain blocks per site thus including all habitat types to account for the endemic species found in the moist forests, upland grasslands, and rocky subtracts.

### Primary forest loss since 2000

We estimated forest loss using a Global Forest Change dataset provided by the Global Land Analysis and Discovery (GLAD), University of Maryland^93^. These data have reasonable accuracy in large, dense evergreen forests, but accuracy decreases for seasonal forests, at low canopy density, and for earlier years (2001–2010). Loss rates are relative to estimated tree cover extent for the year 2000, including plantations. For these reasons, we restricted the estimates of forest loss to an estimate of primary humid tropical forest extent^92^. Nonetheless, we note that there may be inaccuracies owing to the global nature of these forest segmentation/classification algorithms, and that losses in secondary/disturbed are potentially higher.

### Supplementary Information

Supplementary Table S1.

Supplementary Table S2.

## Data Availability

The raw data used in this study are provided in the supplementary materials, including endemic species data and site listings (Table SI1), a table of estimated divergence dates (Table SI2), and spatial data files for the delineation of core sites and convex hull (available at 10.6084/m9.figshare.24586941).
