# Supplementary material for: A biogeographical appraisal of the threatened South East Africa Montane Archipelago ecoregion
Source: Sci Rep. 2024 Mar 12;14:5971. doi: 10.1038/s41598-024-54671-z (PMC10933300; doi:10.1038/s41598-024-54671-z)
Supplement: Supplementary file 2 — Supplementary Table S2. [file 41598_2024_54671_MOESM2_ESM.docx]

# Supplementary Information file: Table SI2. Estimated Divergence Dates of select species and groups.

A biogeographical appraisal of the threatened South East Africa Montane Archipelago ecoregion

| **Group** | **Genus/Species** | **Estimated divergence date** | **Reference Source #** |
| --- | --- | --- | --- |
| Bats | *Rhinolophous* *mabuensis* | 1-2 Mya | 38 |
| Snakes | *Atheris mabuensis* | 15 Mya | 33 |
| Chameleons | *Rhampholeon* spp. | 6-20 Mya | 37, 87 |
| Amphibians | *Nothophryne* spp. | 7.5-18 Mya | 85 |
| Butterflies | *Cymothoe baylissi* | 4 Mya | 49 |
| Crabs | *Potamonautes* and *Maritonautes* | 2.5-8 Mya | 50 |

Table SI2. Estimated Divergence Dates of select species and groups.
